# Two different motor learning mechanisms contribute to learning reaching movements in a rotated visual environment

**DOI:** 10.12688/f1000research.3676.2

**Published:** 2014-12-10

**Authors:** Virginia Way Tong Chu, Terence David Sanger

**Affiliations:** 1 Department of Occupational Therapy, University of Illinois at Chicago, Chicago, IL 60612, USA; 2 Departments of Biomedical Engineering, Neurology, and Biokinesiology, University of Southern California, Los Angeles, CA 90089-1111, USA

**Keywords:** motor learning, adaptation, visual rotation, reaching

## Abstract

Practice of movement in virtual-reality and other artificially altered environments has been proposed as a method for rehabilitation following neurological injury and for training new skills in healthy humans.  For such training to be useful, there must be transfer of learning from the artificial environment to the performance of desired skills in the natural environment.  Therefore an important assumption of such methods is that practice in the altered environment engages the same learning and plasticity mechanisms that are required for skill performance in the natural environment.  We test the hypothesis that transfer of learning may fail because the learning and plasticity mechanism that adapts to the altered environment is different from the learning mechanism required for improvement of motor skill.  In this paper, we propose that a model that separates skill learning and environmental adaptation is necessary to explain the learning and aftereffects that are observed in virtual reality experiments.  In particular, we studied the condition where practice in the altered environment should lead to correct skill performance in the original environment. Our 2-mechanism model predicts that aftereffects will still be observed when returning to the original environment, indicating a lack of skill transfer from the artificial environment to the original environment. To illustrate the model prediction, we tested 10 healthy participants on the interaction between a simple overlearned motor skill (straight hand movements to targets in different directions) and an artificially altered visuomotor environment (rotation of visual feedback of the results of movement).  As predicted by the models, participants show adaptation to the altered environment and after-effects on return to the baseline environment even when practice in the altered environment should have led to correct skill performance.  The presence of aftereffect under all conditions that involved changes in environment demonstrates separation of environmental adaptation and skill learning. Our results support the existence of two distinct learning modules with different adaptation properties.  Therefore we suggest that adaptation to an altered environment may not be useful for training new skills.

## Introduction

Experiments in haptic ( Gandolfo *et al.*, 1996; Patton *et al.*, 2001; Shadmehr & Mussa-Ivaldi, 1994) and virtual-reality environments ( Krakauer *et al.*, 1999; Pine *et al.*, 1996) have repeatedly shown that movement will be altered by changes in environment, and may remain altered for a short time after the original environment is restored (“motion aftereffect”). ( Della-Maggiore *et al.*, 2004; Thoroughman & Shadmehr, 2000) This observation has led researchers to suggest that either the original adaptation or motion aftereffect could be used to train skills ( Rose *et al.*, 1996; Sveistrup, 2004). Unfortunately, in most cases, the effect of the altered environment is only temporarily maintained, and thus there is no transfer of learning from the altered movement to normal skill performance ( Kozak *et al.*, 1993). The lack of transfer between experimental conditions in virtual reality perturbations could be explained by the specificity of learning framework ( Henry, 1968), which stated that transfer of learning depends on the specificity of the conditions of practice. Evidence supporting this framework stemmed from low correlations in skilled performance in similar tasks requiring similar motor skills ( Bachman, 1961; Lotter, 1960). These studies suggested poor generalization of motor skills and conditions that facilitate transfer of learning appear to be very complex and task specific ( Proteau *et al.*, 1992; Wulf & Shea, 2002). We hypothesize that the reason for the lack of transfer in adaptation tasks is that task learning and environment adaptation are performed by two separate learning systems.

Shadmehr and colleagues ( Smith *et al.*, 2006) proposed that there are two learning systems of different time scales that underlie motor learning. These two learning systems are characterized by their time properties. The fast system responds strongly to error but also forgets rapidly, while the slow system responds weakly to error, but retains information. Recent evidence ( Chen-Harris *et al.*, 2008) suggests that the fast system has the structure of a forward internal model and the slow system could be a motor command generator. The adaptation to visuo-motor perturbations has been shown to depend on the cerebellum, and is driven by the sensory prediction error rather than the motor error ( Tseng *et al.*, 2007).

We suggest that these two systems can be separated based on their training signals rather than on their time scales. ( Mazzoni & Krakauer, 2006) separated explicit cognitive strategies and implicit environmental adaptation in an experiment that tested the use of cognitive strategies to counter a visual rotation in a reaching task. Their results showed that implicit motor adaptations override explicit cognitive strategies, demonstrating the interactive nature of the two systems. In this paper, we present a mechanistic explanation through the use of a computational simulation. In particular, we suggest a system that responds to sensory prediction error and learns the structure of the sensory-motor dynamic environment (“fast system”), while another system responds to task performance error and learns the elements needed to perform a task (“slow system”). We further suggest that the two systems have very different generalization properties, so that while the sensory prediction error system can generalize broadly across the environment, the performance error system does not generalize to dissimilar tasks. These properties are consistent with two different and simultaneously-active learning systems, and we will simulate a simple model of this structure to compare with human data. The proposed structure is similar in spirit to a model originally proposed by Doya and colleagues, in which there are separate neuroanatomical regions for motor planning and for adaptation to changes in dynamics ( Doya *et al.*, 2001).

Many experiments that use altered visuo-motor environments confound the two types of error, so that performance error is caused by sensory prediction error. In such cases it is not possible to distinguish the two learning systems. In order to distinguish the two systems we need to test the effect of sensory prediction error when performance error is zero, and the effect of performance error when sensory prediction error is zero. By doing so, we will show that the two systems have very different generalization properties, and therefore cannot be implemented by the same network. Recent studies have distinguished the two mechanisms into model-based learning and model-free learning ( Haith & Krakauer, 2013; Huang *et al.*, 2011). Using modified visuomotor rotation experiments, Krakauer and colleagues showed the contribution of model-free learning in explaining faster relearning of visuomotor rotations. We build on this knowledge and further distinguish the two types of learning by the error that is used by each learning mechanism.

We use a very simple experimental paradigm. The “skill” that we test is the ability to make straight reaching movements to different targets on a pen tablet. This is a very simple and overlearned skill, but it provides a sufficient model for testing the hypothesis. Here we consider movements to different targets to represent different skills, since different movement directions require significant changes in the pattern and timing of muscles used. The “environment” that we test is the relation between hand movement on the pen tablet and the visual image of movement that is seen by the subject. Different rotations of the displayed hand movement with respect to the true hand movement are considered to be different sensory-motor environments.

**Figure 1.**
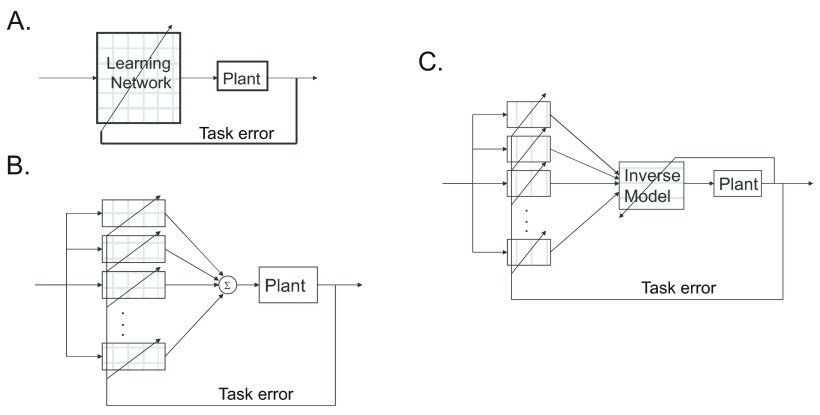
Different neural network models. **A**. Structure (1) is a single learning network that adapts to both changes in skill and environment. The same system learns based on errors in the observed task. **B**. Structure (2) is a motor program model. Learning is performed by separate network for each task based again only on observed error. **C**. Structure (3) is a two system-learning model. This model splits into two groups of systems, where there is a separate system for each task, learning based on observed error, but a single inverse model for control of the environment, learning based on prediction errors.

We compare the results to three simple model structures for skill learning and environment adaptation ( Figure 1). Structure 1 consists of a single network, and structures 2 and 3 have increasingly more complex structure. Each has different generalization properties. **Structure 1**: Skill learning and environment adaptation are performed by a single shared network for all tasks (directions of hand movement) and all environments (visual rotations). This structure predicts that adaptation to a new environment will change performance on multiple targets. It also predicts that practice on one target will affect performance on other targets even without a change in environment. Thus both the environment and the task will generalize across multiple targets, and environment learning will have a broad effect on task learning. **Structure 2**: Task learning and environment adaptation are performed by a single distinct network for each target. This structure predicts that adaptation to a new environment will change performance only on the particular target practiced in that environment. Thus neither the environment nor the task will generalize across multiple targets, but environment learning will have a focused effect on task learning. **Structure 3**: Task learning is performed by a separate network for each target, but environment adaptation is performed by a single shared network. This structure predicts that adaptation to a new environment will change performance on multiple targets, but practice on one target will not affect performance of other targets. Thus the environment will generalize across multiple targets, but the task will not, and environment learning will have no effect on task learning because it is performed by a completely different subsystem. Note that we do not test the fourth implied possibility, in which the task generalizes across multiple targets but the environment does not, because this is not consistent with known previous results ( Goodbody & Wolpert, 1998). Only structures 1 and 2 could permit environment adaptation to be useful for training tasks, since only in these structures are the parameters modified by environment adaptation also used for tasks (see Figure 1).

In this study, we will reject structure 1 by showing that learning one task does not lead to aftereffects on a second task, and therefore show that sufficiently different tasks do not share parameters and are probably learned by distinct networks. Furthermore, we will reject structure 2 by showing that learning a new environment does lead to aftereffects when the environment returns to baseline, and therefore that multiple environments are learned by a single network with a single set of parameters. Our results support structure 3 by showing that learning one environment leads to aftereffects in a different environment, but learning one task does not affect learning of another task. Since adaptation to sensory-motor error generalizes broadly while task learning generalizes narrowly, we claim that environment learning and task learning cannot be implemented by the same network. An interesting consequence of the independence of the two systems is that when the task error is zero but the sensory-motor mismatch is nonzero, adaptation reduces the mismatch even at the expense of worsening the task error, confirming the results of ( Mazzoni & Krakauer, 2006). Therefore adaptation to the environment is controlled independently of the task error, and our results will support the existence of two different learning systems that respond to two different types of error.

Motion aftereffect paradigms have provided useful results concerning generalization of adaptation to different task or environment parameters. ( Krakauer *et al.*, 1999; Shadmehr & Moussavi, 2000; Vetter *et al.*, 1999; Krakauer *et al.*, 2006; Hwang *et al.*, 2006) Here, we show that the type of generalization depends on the type of error that drives learning, consistent with the hypothesis of two different learning systems that are distinguished by the error to which they respond and the way in which they generalize across the environment.

## Simulations

One example of each of the three models was simulated. To do this, several assumptions were made. We assumed that the participants had already learnt the dynamics of their arm and know the motor commands for reaching movements. Therefore, the learning of the arm dynamics was not included in the model. Furthermore, we assumed that the participants would have already learnt what trajectory would solve the problem optimally from previous experience. Based on physiological studies, human movements are observed to have a bell-shaped velocity. ( Gordon *et al.*, 1991; Bizzi & Abend, 1983). This type of velocity profile has been shown to be optimal for many cost criteria such as the minimum-jerk criterion in optimal controlled reaching ( Nagasaki, 1989). For simplicity, the optimal controller (trajectory generator) was modeled with a desired trajectory generator, which generates a straight-line trajectory to the target with a bell-shaped velocity profile. The bell-shaped profile used is a normalized truncated Gaussian distribution function, which is lowered so that it has zero initial velocity.

### Model components

**Figure 2.**
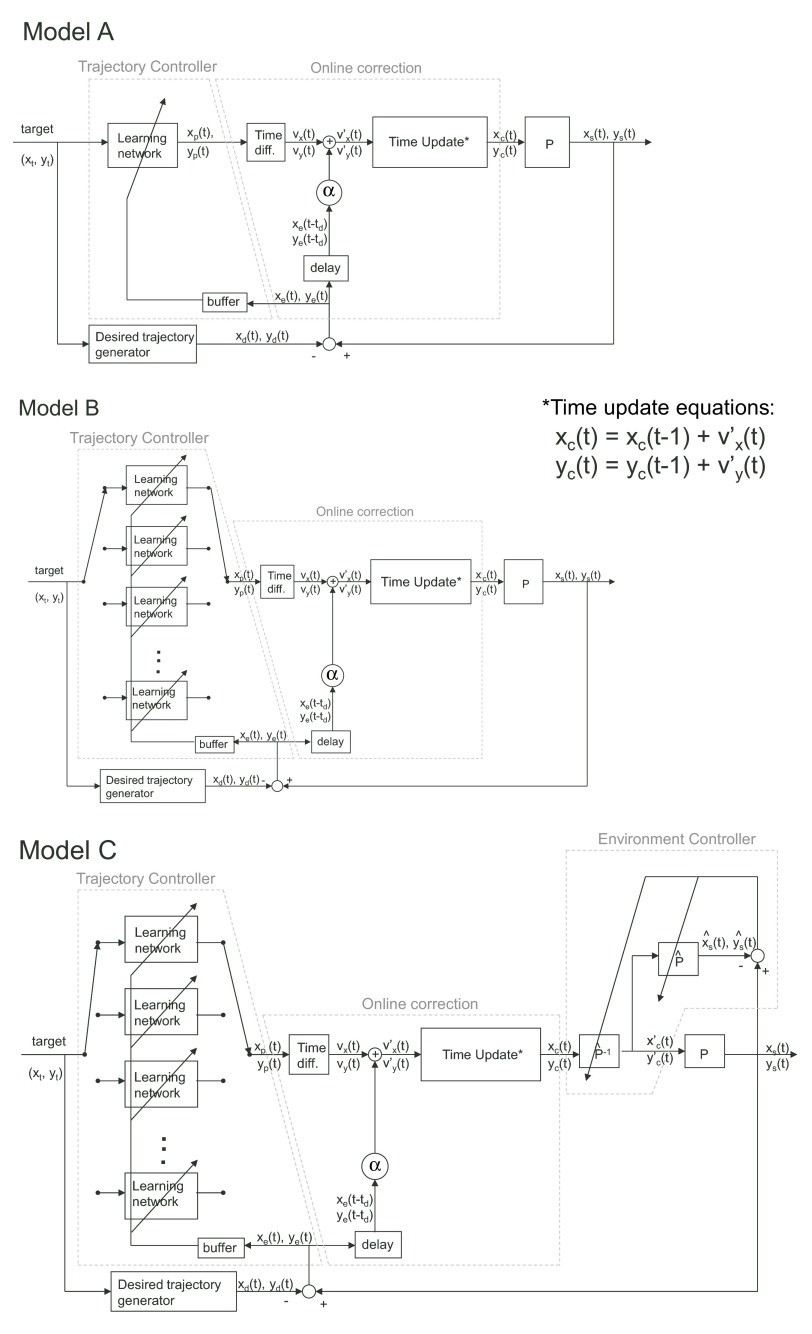
Block diagram of the three simulated models. P (Plant) is the matrix that represents the rotation of the visual feedback. P ^-1^ is the internal inverse model of the plant. The desired trajectory generator uses a bell-shaped velocity profile to generate the desired trajectory. The “Time diff” block calculates the velocity sequence that would have produced the planned trajectory using the planned position sequence. Using the planned velocity sequence as the input command, the online correction takes the delayed error as a proportional correction term to give the new velocity to guide where the cursor should go relative to the current location. The learning networks in the models are basis function (BF) networks where each basis function is an increasing order monomial of time. **A**. Detailed simulation model for model **A** (simple learning model). **B**. Detailed structure for model **B** (motor program). **C**. Detailed structure for model **C** (two-system learning model).

There were three components used in the models: the trajectory generator, the environment adapter and the online feedback controller. The use of these elements is illustrated in Figure 2. From Figure 2 we see that in models A and B there is one environment adaptation module for every trajectory generation module. Therefore, in the simulations of models A and B we used the same network for trajectory generation and adaptation to the environment. Model A has a single network for trajectory generation and environment adaptation, while model B has a separate network for each task that learns both the trajectory and the environment. The separation of learning networks for each task is a simplification in modeling to replicate a task generator that generates a command trajectory for each desired target in a continuous fashion. With this simplification, we could use basis function networks to model task learning rather than other more complicated networks. In model C, there is a separate trajectory generator for each task but only a single environment adaptation module, so two different types of network must be used. In the trajectory generator module, the error signal is based on performance error, the error observed by the participant during each trial. For the environment adapter, the error that trains the network is based on the prediction error, the difference between the participant’s anticipation of movement and the actual observed movement.

For any task k, the desired trajectory generator creates a desired trajectory x ^[k]^_d_(t) and y ^[k]^_d_(t). This trajectory is compared with the observed feedback to generate the error signals x _e_(t) and y _e_(t). At each trial, the learning network produces the planned trajectory, x _p_(t) and y _p_(t). x _p_ and y _p_ are the input to a feedback controller. The feedback controller combines the planned trajectory and the error x _e_(t) and y _e_(t) at each point in movement in order to generate a motor command x _c_(t) and y _c_(t). The plant takes the motor command x _c_(t) and y _c_(t) (which is just a desired position on the pen tablet) and transforms it into the observed position x _s_(t) and y _s_(t) that is then available as sensory (visual) information. The plant transforms the motor command into the sensory output by rotating, so that [xsys]=P(θ)[xcyc], where P(θ) is a 2×2 rotation matrix.

$$P\left(\theta \right)=\left[\begin{array}{c} cos\theta \\ sin\theta \end{array}\;\begin{array}{c} -sin\theta \\ cos\theta \end{array}\right]$$

The sensory output is compared with the desired trajectory to generate the error signal x _e_ = x _d_ - x _s_, y _e_ = y _d_ - y _s_. The error signal is used in real-time for feedback control, and it is used at the end of each movement attempt to update the trajectory generator module. Assuming that the desired trajectory (x _d_, y _d_) is known, the goal of the trajectory generator is to minimize the cost function, ε, the norm of the error signal integrated over time.

$$\varepsilon =\int \left\|\left[\begin{array}{c} x_{e}(t)\\ y_{e}(t) \end{array}\right]\right\|dt$$

In addition to the above elements, model C includes a separate environment adaptation module that modifies the motor command x _c_ y _c_ and changes it to x′ _c_ y′ _c_ in order to compensate for changes in the plant (rotation). It performs this modification by using a plant inverse *P* ^–1^( *θ*) that predicts the correct motor command x′ _c_ y′ _c_ for any desired sensory output x _s_, y _s_. The plant inverse is learned by approximating the plant “forward model” from x′ _c_ y′ _c_ to x _s_, y _s_ and then inverting the resulting 2×2 matrix. Note that in models A and B, a change in the environment results in a change in the trajectory generator because of the increased performance error x _e_ and y _e_. In model C, a change in the environment will be compensated by the environment adaptation module and thus the trajectory generator module will not change.

### Trajectory generator

The input to the trajectory generator was the x and y coordinates of the target location (x _t_, y _t_) as shown on the display. Using the given target (x _t_, y _t_), the desired trajectory generator generated a trajectory (x _d_(t), y _d_(t)) that it “hopes” to see on screen, based on the assumptions mentioned above (straight and bell shaped velocity).

The learning network was programmed as a basis function neural network. The basis functions used were polynomials of time up to degree n. Let W _x_ ∈ ℜ ^n^ and W _y_ ∈ ℜ ^n^ be unknown weight vectors. Then the output of the trajectory generator was written as

    x _p_(t) = Σ _i_ W _xi_ × Φ _i_(t)    and    y _p_(t) = Σ _i_ W _yi_ × Φ _i_(t)

where W _xi_ and W _yi_ were the i ^th^ elements of the W _x_ and W _y_ vectors and Φ _i_(t) is the i ^th^ degree monomial of t (Φ _i_(t) = t ^i^). These weighted basis functions were passed through a summation operator to sum across all i ^th^elements. The outputs x _p_(t) and y _p_(t) were then passed to the rest of the learning model. The outputs x _p_(t) and y _p_(t) specify the trajectory input provided to the feedback controller.

The weight vectors W _x_ and W _y_ were trained using the errors (x _e_(t), y _e_(t)) between the desired trajectory (x _d_(t), y _d_(t)) and the trajectory of the movement observed on the screen (x _s_(t), y _s_(t)). The training algorithm is the Widrow-Hoff “least mean squares” (LMS) training algorithm that is known to converge for stationary inputs. ( Widrow & Hoff, 1960; Widrow *et al.*, 1976). Unfortunately, when used in a control system, the inputs are non-stationary and thus convergence of LMS is not guaranteed. Nevertheless, this is a commonly used algorithm that has been shown to perform well in adaptive control tasks ( Sanger, 1991; Sanger, 1994; Sanner & Slotine, 1992) and it provides one of the simplest models of motor learning ( Berthier *et al.*, 1993; Schweighofer & Arbib, 1998).

    x _e_(t) = x _d_(t) – x _s_(t)         and     y _e_(t) = y _d_(t) – y _s_(t)

    ∆W _xi_ = λ Σ _j_ x _e_(t _j_)Φ _i_(t _j_)   and    ∆W _yi_ = λ Σ _j_ y _e_(t _j_)Φ _i_(t _j_)    for all i

    W _x_ = W _x_ + ∆W _x_            and     W _y_ = W _y_ + ∆W _y_

λ was the learning rate of this system, i was the degree for the basis function described above, and j was the time index used for the summation of errors from the whole movement. Each weight (W _xi_ and W _yi_) was updated after each trial, based on the errors from the whole movement. In models A and B, the output x _p_ and y _p_ represents both task learning and adaptation to the environment, since a change in the environment or the desired trajectory will lead to a change in error x _e_ and y _e_ that will modify the weights in the network. In model C, there is a separate stage of environment adaptation and thus this initial network is responsible only for adapting to changes in the task. In models B and C, the multiple neural network structure was simulated by storing multiple weight vectors W(k) _x_ and W(k) _y_ which can be trained or retrieved when needed for any particular task k.

### Environment adapter

The environment adapter built an internal model of the environment, $\hat{P}$, giving predictions, ${\hat{x}}_{s}$, ${\hat{y}}_{s}$. The internal model was inverted to provide the environment inverse ${\hat{P}}^{–1}$. This gave the learning system a way to anticipate the rotational field and attempted to “undo” its effect. At each trial, the input to the environment inverse was the output from the feedback controller (x _c_(t), y _c_(t)). The model inverse produced the modified command trajectory (x′ _c_(t), y′ _c_(t)) that the system anticipated could invert the plant. This signal is then fed through both the plant and the plant model. By computing the error between the plant output and the plant model output (e _p_), the plant model $\hat{P}$, could be trained. Training of the plant model was done through the LMS algorithm. Since the posed problem was essentially solving a linear regression, the LMS is guaranteed to converge, assuming that the learning rate is not too large.

$$e_{p}=\left[\begin{array}{c} x_{s}\\ y_{s} \end{array}\right]-\left[\begin{array}{c} {\hat{x}}_{s}\\ {\hat{y}}_{s} \end{array}\right]\;\;\;\;\Delta \hat{P}=\alpha_{e}e_{p}{\;[\begin{array}{c} x{'}_{c}\\ y{'}_{c} \end{array}]}^{T}\;\;\;\;\left[\begin{array}{c} x{'}_{c}\\ y{'}_{c} \end{array}\right]=\hat{P}{}^{-1}\;\left[\begin{array}{c} x_{c}\\ y_{c} \end{array}\right]$$

where α _e_ was the learning rate of the system.

### Online feedback correction

As a human participant would, the system in the simulation should also be allowed to correct errors online. Therefore, an online feedback correction controller was implemented. The planned trajectory (x _p_, y _p_) from the trajectory planner was used as input to the controller and it was also given a feedback of the error “observed” on screen (x _s_, y _s_). This error was calculated based on the desired trajectory (x _d_, y _d_). The online feedback is delayed by 100ms, the same order of magnitude of recorded human visual reaction time. ( Fischer & Ramsperger, 1984) The controller output was proportional control using the delayed feedback with a feedback gain γ, added to the feedforward control generated using x _p_ and y _p_.

    v _x_(t) = x _p_(t) – x _p_(t-1)       v _y_(t) = y _p_(t) – y _p_(t-1)

    v’ _x_(t) = v _x_(t) + γx _e_(t-t _d_)       v’ _y_(t) = v _y_(t) + γy _e_(t-t _d_)

    x _c_(t) = x _c_(t-1) + v’ _x_(t)      y _c_(t) = y _c_(t-1) + v’ _y_(t)

## Experimental methods

In a visual rotation (VR) setting, there are two aspects to any task: vision (what the participant sees) and motor (the participant’s actual movement). In our experiment, the participants observed the visual feedback of their movement on a LCD monitor and performed the movement using their unseen hand under the monitor. We manipulated the relationship between movement and visual feedback in order to force the participants to adapt to a new motor-sensory map (environment). We determined whether this adaptation interfered with performance of a previously-learned task (straight line movements in different directions). In one experimental manipulation, participants were asked to make a movement that appeared visually the same, for example, moving toward the same target on the screen, but required a different hand movement due to a change in the visual-motor map. We will refer to this as “same task (target) different environment (visuo-motor map)”. In a second experimental manipulation, we asked the participant to make a movement to a different target but without a change in the visual-motor map. This required a change in the actual (unseen) hand movement, so we refer to this as “different task same environment”. In a third experimental manipulation, we asked the participant to make a movement to a different target, but the visual-motor map was changed so that successful performance occurred for the same hand movement in both cases. In other words, the change in target and the change in visual-motor map were in exactly opposite directions and cancel each other out. We refer to this case as “different task different environment”, although the required hand movement did not change. The movements were recorded using a pen tablet (Wacom, Intuos 2 XD-0912-R, Saitama, Japan) connected to a personal computer (Fujitsu, Lifebook T4010, Tokyo, Japan). The participants were asked to complete four short experiments. In each experiment, there were three blocks of 20 reaching trials, reaching from the center of the screen to a target location on a circle. The first and third block were always under the same experimental condition and in the second block, we changed the vision and/or the motor aspect of the task. The experiment was designed to determine whether the condition in the second block interfered with performance of the skill attempted in the first block by causing aftereffects at the beginning of the third block. Thus the three blocks are namely, baseline, interference, and re-adaptation. The outcome measure was a comparison between baseline (before the interfering condition) and re-adaptation (after the interfering condition). The four experiments were carried out in a pseudorandom order for each subject, such that each subject completed all 4 experiments in a randomized order to minimize the effect of the familiarization of the task on any particular experimental condition.

Since straight-line reaching is a heavily practiced skill for most subjects, the visual environment was rotated 10 degrees clockwise in the baseline condition. The baseline condition was therefore no more familiar to subjects than the interference conditions, so subjects were unable to use their extensive prior experience with reaching to override errors induced by the adaptation. Changes in the rotation feedback (environment) were made relative to the baseline environment.

### Procedure

**Figure 3.**
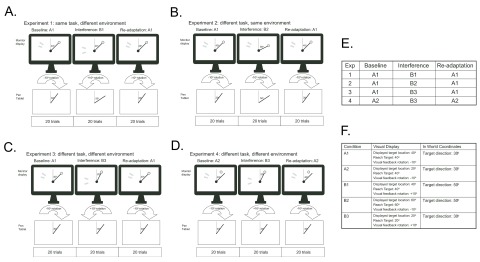
Experimental procedure. In each of the four experiments, there were three blocks, with 20 trials each. In the figure, the monitor represents what the participants saw during the experiment. The rectangle below represents the subjects’ arm movement. The dark circular dot represents the starting location, and the light colored dot represents the target presented to the participants during the experiment. The line in between the dots indicates the desired movement as observed on screen. Two striped marks on the side of the screen always indicate the “west” direction on the tablet, which was used as an indicator to the participants to let them know which environment they are in. Figures **A**, **B**, **C** and **D** show the experimental setup for experiment 1, 2, 3 and 4 respectively. **E** and **F** show details of each experimental block.

In experiment 1 (same task, different environment), the feedback of the movement in the interference block was rotated counterclockwise by 20° relative to baseline (see Figure 3). The target location on the visual display was not changed. This was a typical aftereffects paradigm, where the subject was asked to perform the task with the same specification, but with a change in environment. Two things changed in the experimental condition between the first and second block of the experiment: the visuo-motor map and the movement the subject was required to make. In order to tease apart which is the main cause of the aftereffects, we designed experiment 2 and 3 to test each aspect. In experiment 2 (different task, same environment), the visual feedback was not rotated, but the target location moved in the interference block. This experiment was designed to look at interference between learning different hand movements. In experiment 3 (different task different environment), the feedback of the movement in the interference block was rotated counterclockwise by 20° relative to baseline. The target location (task) in state B was also rotated by the same amount in the opposite direction to keep the required arm movement the same. This experiment was designed to look at the effect of changes in the visuo-motor map (environment) without a change in the hand movement required to solve the problem. However, in order to test, the target location had to be different between the baseline and interference conditions. Therefore, a fourth experiment, Experiment 4 (different task, different environment) was designed as a control for experiment 3 where the visual display was not changed throughout the three blocks. The purpose of this control is to ensure that the effects in experiment 3 were not simply due to the altered sensory display. In the baseline condition, instead of asking the subjects to reach to the target location, we placed the target at 20°, and asked the participant to reach to 40°. Additional feedback was given in form of a score. The score was calculated as 100 - the target error (in degrees). The target error was calculated at the point when the subject’s hand/cursor crossed the circle where the target lies. In the interference block, the target was still kept at 20° and the feedback of the movement was rotated counterclockwise 20°. The participants were asked to reach to the target to keep the movement the same. Therefore, the visual display did not change although the target and environment did. We used this experiment to verify whether the results observed in experiment 3 could be explained by changes in the visual display and to separate the effects of changes in the intended movement from changes in sensory stimulus (visual display).

Because subjects may make large errors following an unexpected change in the visual rotation, they were provided with a visual indication of the environment, and this indication also served as a warning when a change occurred. The indicator on screen marked the “west” direction on the pen tablet. Therefore, when the visual feedback was rotated, the indicator moved on the screen according to the rotation field. The participants were instructed to reach out from the center as fast and as straight as possible. If they completed the movement and reached the target within a time limit (1 s), the target would flash orange to indicate a success. After each trial, the participant was guided to move the cursor back to the starting location without direct feedback of the cursor location. On the screen, the participants were shown a circle whose radius represents the distance between the subject’s cursor and the starting location. The participants were told to move their cursor to minimize the size of the circle in order to move the hidden cursor back to the starting point.

### Participants

Ten participants with no history of neurological diseases were recruited from Stanford University. The participants were between the age of 23 and 27, six males and four females. All participants were right-handed and performed the experiments with their right hand. Follow-up tests with another group of participants were carried out after the conclusion of the first study. The follow-up study was on the same rotation paradigm only with larger changes in rotation angles. For the follow up study, eight adult participants (average age: 25.4, five females and three males) were recruited for further tests. These eight participants did not participate as a subject in the first group. All except one of the follow-up study participants were right handed. All participants performed the tasks with their dominant hand. All procedures were approved by the Stanford University Institutional Review Board. Participants signed written consent for the experiment and HIPAA authorization for the use of personal data.

### Data analysis

Data analysis was performed in MATLAB. The primary data were the samples of the pen tablet position sampled at 50Hz. The initial direction of movement was calculated as the angle of the line connecting the start location to the point of maximum velocity ( Krakauer *et al.*, 1999). For each trial, the average and standard deviation of the initial reach angle for all participants were calculated. The extent of an aftereffect in each experiment was determined by comparing the first trial in state C with the baseline statistics computed from the last ten trials of state A. The baseline statistics, means (μ) and standard deviations (σ), were calculated for each experiment per participant. Using the measurement from the first trial of state C (x _c1_), the magnitude of the aftereffect was calculated as a z-score.

$$Z=\frac{x_{c1}-\mu }{\sigma }$$

A larger z value is associated with a greater aftereffect. The presence of an aftereffect was tested statistically by performing a hypothesis test with α = 0.05. When |z| > 1.96, the null hypothesis (x _c1_ belongs to the baseline distribution) was rejected, and we asserted the presence of a statistically significant aftereffect. Furthermore, we compared the magnitude of the aftereffects to the baseline trials and between experiments. The last trial in state A was compared to the first trial in state C for all 4 experiments using repeated measures ANOVA. The initial direction was the dependent measure, and the experiment (1 to 4) and the state (A and C) were the independent measures. Post-hoc pairwise statistics were performed using Fisher protected least square difference tests.

## Results

### Simulation results

Before the simulation of the experiment, the model was allowed to “practice” straight lines from the center to the various target location, without the rotation of the “visual feedback” (not shown in the figure). The models practiced several hundred straight lines movements to targets at 0° (straight up), 20°, 40° and 60°. This allowed the model to have relatively similar experience as a human subject, where humans are assumed to already know how to make straight lines to the various targets in the normal environment. The learning rates in the models were tuned by starting with very small values, and increasing them until the system was able to learn in approximately the same speed as the human subjects (within 20 trials). The online feedback gain was tuned to adjust the trajectories such that the trajectories would end at the target location. The system was approximately overdamped even if there was large initial error.

The simulations showed a small learning curve at the beginning of experiment 1. This was because the model has to learn to adapt to the +10° rotational field. The simulation started with doing experiment 1 and we did not program a break in between the experiments, so the computer could retain the +10° field learnt from before and did not show a learning curve in the beginning of experiment 2 and 3.

**Figure 4.**
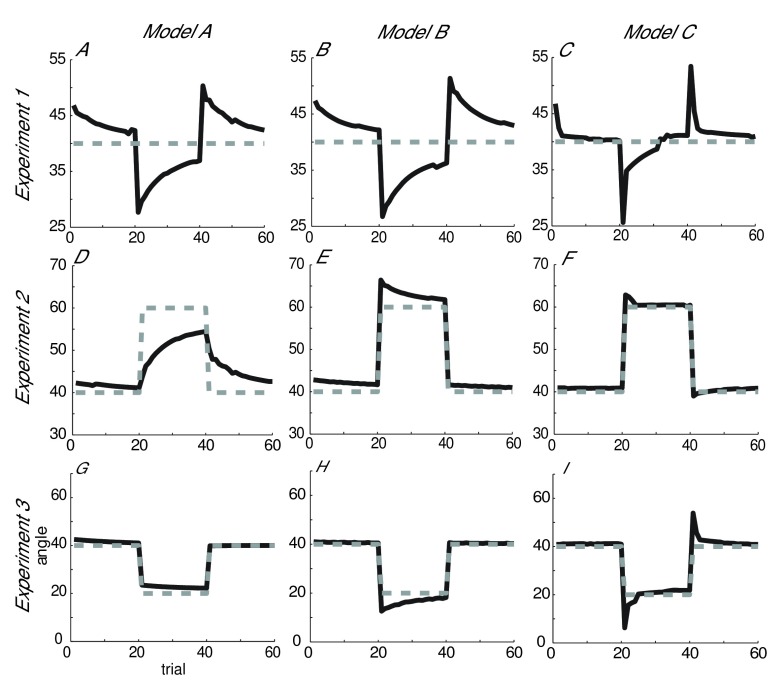
Results from one simulation run of each model. Results plotted are the initial angle (in degrees) of the simulated movement observed on screen against the trial number in that experiment. The solid black line represents the computer simulation results; whereas the dotted grey line represents the target presented for that trial. Experiment 4 was not simulated due to the nature of the experiment being very similar to experiment 3. To a computer simulation, experiment 3 and experiment 4 are the same as the difference between the two experiments comes from difference in visual display. The first column ( **A**, **D**, **G**) are the results from model **A**, second column ( **B**, **E**, **H**) are results from model **B** and the third column ( **C**, **F**, **I**) are from model **C**. The first row ( **A**, **B**, **C**) are results for experiment 1, the second row ( **D**, **E**, **F**) are results for experiment 2, and the third row ( **G**, **H**, **I**) are results for experiment 3.

Our simulation results highlighted the following model predictions for each structure: Model A (Structure 1) predicted that practice on one target will affect performance on other targets even without a change in environment ( Figure 4D). Model B (Structure 2) predicted that adaptation to a new environment will change performance only on the particular target practiced in that environment ( Figure 4H). Model C (Structure 3) predicted that adaptation to a new environment will change performance on multiple targets ( Figure 4I), but practice on one target will not affect performance of other targets ( Figure 4F).

### Human participant experimental results

According to the calculations outlined earlier (results shown in Table 1, raw data in dataset 1), in experiments 1 and 3, ten participants showed significant aftereffects that reached statistical significance (≥ 0.05); in experiment 4, nine participants showed aftereffects. In experiment 2, only three participants showed a significant aftereffect. In experiment 1, all subjects showed transient aftereffects both at the onset of the altered environment (first few trials of state B) and at the return to the baseline environment (first few trials of state C). This agrees with previous results and shows that subjects adapted to the altered environment in a way that suggests the presence of an adaptive internal model ( Kawato, 1999; Wolpert *et al.*, 2001). Aftereffects were also seen in experiment 3 and 4. Note however, that although subjects achieved the desired performance in the altered environment (at the end of state B), the return to the baseline environment caused worsening of performance (beginning of state C) that only gradually returned to its original baseline. Aftereffects were not observed in experiment 2.

**Table 1.** Statistics of the aftereffect. Represented in the table are the aftereffect z scores calculated for each participant for the 4 experiments (Exp). The bolded numbers are the ones considered significant under the assumption of α=0.05 (|z| > 1.96). The significant column (Sig.) in the table represents the number of z scores (out of 10) that are significant in that experiment. The table also includes results from the three models: + indicates a presence of aftereffects prediction and – indicates an absence of aftereffects prediction.

Exp	Human participants											Model prediction		
	1	2	3	4	5	6	7	8	9	10	Sig.	A	B	C
1	**6.30**	**4.85**	**4.64**	**9.82**	**2.50**	**3.66**	**6.91**	**10.9**	**8.05**	**15.4**	10	+	+	+
2	1.48	1.81	1.78	1.40	**5.71**	0.69	-0.28	0.31	**2.30**	**2.20**	3	+	-	-
3	**4.77**	**7.13**	**7.31**	**6.75**	**5.51**	**3.83**	**2.94**	**2.96**	**6.42**	**6.14**	10	-	-	+
4	**2.11**	**2.21**	**10.6**	**5.65**	0.33	**5.04**	**3.52**	**2.12**	**5.28**	**3.54**	9	-	-	+

**Figure 5.**
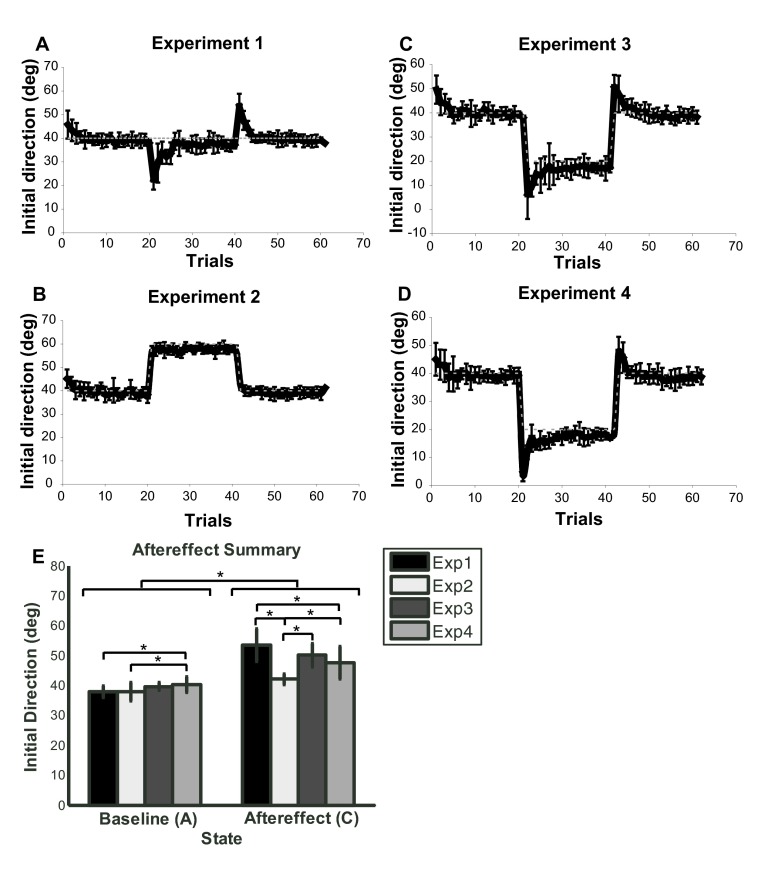
Experimental results of human participants. The initial direction measurement of the movement observed on screen. This is the trial by trial average of all ten subjects. The error bars mark the standard deviation amongst the subjects and the dotted grey line represents the desired target angle observed on screen. The vertical axis is the initial direction measured in degrees and the horizontal axis is the trial number. **A**. Experiment 1, same task different environment. **B**. Experiment 2, different task same environment. **C**. Experiment 3, different task different environment. **D**. Experiment 4, different task different environment (same display target). **E**. Group mean of the last trial in state A (baseline) compared to the first trial in state C (aftereffects) in a bar graph. Asterisks indicate statistical significant difference between groups (p < 0.05).

These results were confirmed using repeated measures ANOVA, examining the difference between the last trial of state A (baseline) and the first trial of state C (aftereffect), with the initial direction as the dependent measure, and the experiment (1 to 4) and the state (A and C) as the independent measures. The initial direction was statistically different between the experiments (F(3,27) = 13.18, p<0.0001) and between baseline and aftereffect (F(1,27) = 376.76, p<0.0001). The interaction between the experiment and state was also statistically significant (F(3,27) = 10.703, p<0.0001). The post hoc Fisher PLSD test showed that initial direction measure in state C (aftereffects) were significantly higher in experiment 1, 3, and 4 compared to experiment 2 (exp 1-2: p < 0.0001, exp 2-3: p = 0.0002, exp 2-4: p = 0.0072, see Figure 5E).

**Figure 6.**
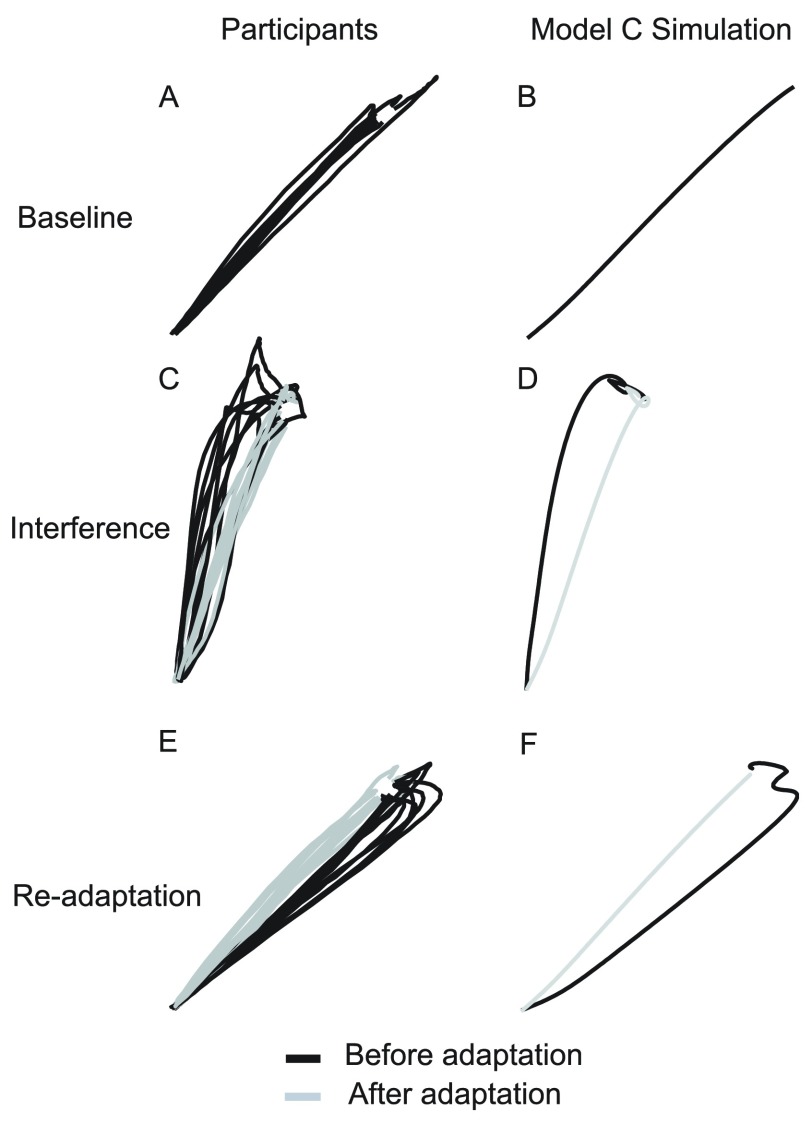
Trajectories of participants along with trajectories from the simulation of model C. The trajectories are taken from Experiment 3. The participants plot contains the trajectories from all ten participants for that particular trial. **A**, **C**, and **E** are from participants. **B**, **D**, and **F** are from simulations. **A** and **B** are one of the baseline trajectories. **C** and **D** are from the interference state. **E** and **F** are from the readaptation state. In figures **C**– **F**, the black lines are from the first trial of the state, and the grey lines are from the last trial of the state.

By comparing Figure 4 and Figure 5, we see that models A and B do not match the experimental results. Only model C is consistent with human results in all of the experiments and predicts aftereffects in experiments 1, 3, and 4, but not 2. Figure 6 shows several typical trajectories from a participant and simulations of model C. The terminal “hook” is due (in the model) to the feedback controlling online correction.

**Figure 7.**
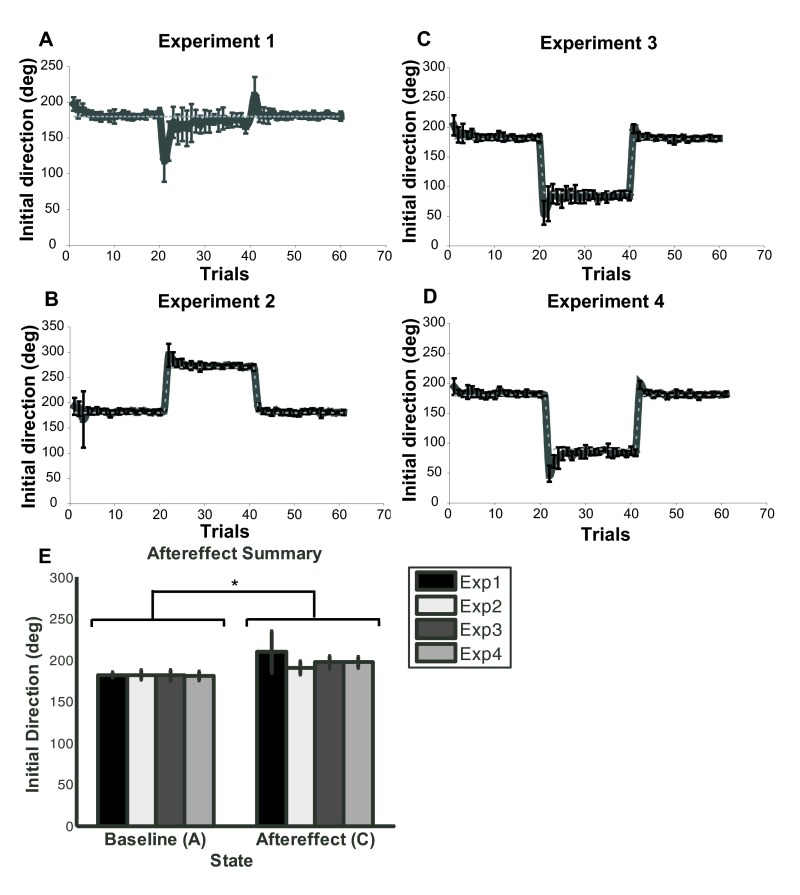
Experimental results for the 90° experiment. The figure shows initial direction measurement of the movement observed on screen. This is the trial by trial average of all eight subjects. The error bars mark the standard deviation amongst the subjects and the dotted grey line represents the desired target angle observed on screen. The vertical axis is the initial direction measured in degrees and the horizontal axis is the trial number. **A**. Experiment 1, same task different environment. **B**. Experiment 2, different task same environment. **C**. Experiment 3, different task different environment. **D**. Experiment 4, different task different environment (same display target).E. Group mean of the last trial in state A (baseline) compared to the first trial in state C (aftereffects) in a bar graph. Asterisks indicate statistical significant difference between groups (p < 0.05).

In the follow-up study, a second group of participants (8 in total) were recruited to study the generalization of aftereffects in larger rotation angles. We repeated our first experiment with a 90° rotation between environments. The targets were also further apart. The targets were located at 90°, 180° and 270° rather than 20°, 40°, and 60° respectively. We call this the 90 degrees experiment. The results in the 90 degrees experiment are presented in Figure 7 and the statistics in Table 2, with the raw data in dataset 2.

**Table 2.** Statistics of the aftereffect in the 90 degrees rotation experiment. Represented in the table are the aftereffect z scores calculated for each participant for the 4 experiments (Exp). The bolded numbers are the ones considered significant under the assumption of α=0.05 (|z| > 1.96). The significant column (Sig.) in the table represents the number of z scores (out of 8) that are significant in that experiment.

Exp	Human participants								
	1	2	3	4	5	6	7	8	Sig.
1	**21.0**	**15.0**	**12.6**	**6.51**	1.92	**11.3**	0.19	**3.43**	6
2	1.63	**3.07**	**5.05**	0.58	-0.32	**6.69**	-0.25	**3.30**	4
3	**2.48**	**4.35**	1.75	**6.33**	**3.64**	**1.98**	**3.99**	**3.12**	7
4	1.17	**3.52**	**3.18**	**2.78**	**4.62**	**3.51**	**9.67**	**4.22**	7

The results in the 90 degrees experiment were not as strong when compared to the original 20 degrees experiment. However, the conclusion still stands that in experiment 1, 3, and 4, the participants showed stronger aftereffects than in experiment 2. The initial errors that occurred in the interference state of experiment 2 showed the environment learner’s limited ability to generalize to targets that are far away from the initial training. Yet the observation that training of a new target in the second environment did not interfere with the performance of the initial target upon return to the re-adaptation state reinforces the separation of task learning and environment adaptation. Aftereffects were observed in experiment 3 and 4 indicating that the training in a different environment at a target 90° away from the initial target interfered with the initial training. The statistical results using repeated measures ANOVA showed that the initial direction was statistically different between baseline and aftereffect (F(1,21) = 53.437, p=0.0002), but no statistical difference was observed between the experiments (F(3,21) = 1.739, p=0.1897, see Figure 7E).

Data from motor learning experiments Dataset 1. Data from original experiment Each column is the initial angle (in degrees) of arm movements for each participant and the model simulation of each trial. The reach target was also shown in degrees. A., B., C. and D. contains data for experiment 1, 2, 3 and 4. Model simulations were only included for dataset 1A, 1B and 1C.
Dataset 2. Data from 90 degrees rotation experiment Each column is the initial angle (in degrees) of arm movements for each participant in the second experiment. The reach target was also shown in degrees. A., B., C. and D. contains data for experiment 1, 2, 3 and 4. Click here for additional data file. Copyright: © 2014 Chu VWT and Sanger TD 2014 Data associated with the article are available under the terms of the Creative Commons Zero "No rights reserved" data waiver (CC0 1.0 Public domain dedication).

## Discussion

Consistent with prior force and rotation field studies ( Gandolfo *et al.*, 1996; Martin *et al.*, 1996; Patton *et al.*, 2001; Shadmehr & Mussa-Ivaldi, 1994), participants showed transient aftereffects when they returned to the original experimental condition after practicing in a different rotational field (experiment 1). However, aftereffects did not occur following a change in the task (experiment 2). Aftereffects did occur in a different rotational field even when the task was adjusted so that the required hand movement did not change between conditions (experiment 3). Aftereffects also occurred in a different rotational field when both the visual display and the required hand movement did not change (experiment 4).

Aftereffects can be considered a type of interference between conditions in which the prior condition affects the initial performance in the subsequent condition. Our results show that interference between conditions occurs if and only if there is a change in the rotational field (the environment). A change in the target task is not sufficient, by itself, to cause interference or aftereffects. Since changes to the environment interfere with each other (experiment 1, 3, and 4), the results suggest that there is only a single environment internal model that adapts and re-adapts (thereby showing aftereffects); whereas there are multiple independent modules for task performance (experiment 2). In this model simulation (Model B and C), we considered each reaching target to have independent but similar learning networks. In reality, this is not realistic as it would require a potentially infinite number of learning networks to model all possible reaching directions. There must be a balance between the ability to switch between tasks quickly and the resources needed for simultaneous storage of multiple learning networks. This is likely accomplished through a structure that is different from our current understanding and requires further research.

The most important observation comes from experiment 3, in which environment adaptation exactly compensated for errors in task performance. In this experiment, adaptation to the rotation of the visual environment caused the hand movement to solve the desired task. Immediately after the visual environment was returned to the baseline, the next attempted hand movement should have low error. If environment adaptation and task learning shared a common mechanism, then the low task error should have resulted in continued good performance. However, as soon as the environment returned to baseline, there was a higher than expected sensory-motor mismatch, and the subjects responded by adapting to the mismatch, even though this resulted in worsening task error. Therefore environment adaptation is not controlled by task performance error. This result is similar to the results of Mazzoni and Krakauer and probably is due to the same mechanism ( Mazzoni & Krakauer, 2006). Our results strongly imply that the two systems use different learning mechanisms. An important consequence is that for this very simple set of tasks, adaptation to an altered environment does not lead to improvements in task performance.

This conclusion is supported by the model simulations. From Figure 4 and Figure 5 we see that model A is not consistent with the human data since there are aftereffects in experiment 2 in the model but not in the human data. Model B is not consistent since there are no aftereffects in experiment 3 in the model but there are in the human data. Only model C correctly predicts the presence of aftereffects in experiments 1 and 3 but not in experiment 2. Model C includes separate networks for each task, but a shared network that adapts to the environment. Thus the match between data and simulations of model C supports our hypothesis.

Although the focus of this study is on typical motor behavior, we can suggest hypotheses about patient deficits using our model simulations. Research has shown that patients with cerebellar disorders have difficulties with environmental adaptation and demonstrate perseverant behavior when switching between environments ( Baizer *et al.*, 1999; Martin *et al.* 1996a; Morton & Bestian, 2006). These patients demonstrate ability to learn a new movement but when introduced to a new environment, they show no adaptation, hence no aftereffects. In some circumstances, there has been early evidence that showed this inability to adapt to environmental differences can be used to temporarily improve movement patterns in these patients ( Malone & Bastian, 2014). The lack of environmental adaptation is similar to the simulation results of model B, demonstrating a lack of aftereffects in Experiment 3. The other difference between our simulation and patients with cerebellar disorder is the learning rate. In our simulation, we used a relatively fast learning rate to simulate typical adult performance, but patients with any movement disorders often have a slower learning rate. Without a separate environmental learning network, there will be no adaptation to changes in the environment and a slower learning rate will require more trials for equivalent performance in this population.

These experimental results are consistent with prior results on generalization ( Krakauer *et al.*, 2000; Vetter *et al.*, 1999). Previous studies concluded that environment adaptation to visuomotor rotation has good generalization properties for targets within 45°. This is consistent with the aftereffects the participants and the model simulations showed in our 20° rotation experiments. However, based on these previous results, aftereffects were not consistently observed for the targets were more than 45° apart from each other. In order to test the generalization of the model to targets more than 45° apart, we performed the 90 degree experiment. If there were no generalization of the environment adaptation to targets more than 45° apart, there should be no aftereffects in experiments 3 or 4, where the participants were trained in a different environment at a target that was 90° away from the target used in the first environment. Since aftereffects were observed in the human subject results in experiments 3 and 4, we infer that environmental adaptation can be generalized to targets larger than 45° apart although the effects are smaller than the 20° rotations. This is consistent with previous research showing that visual rotations between 75° and 120° had smaller facilitation effects, as the brain appears to use different strategies for rotations beyond 90° ( Abeele & Bock, 2001).

An important consequence of these experiments is that training in the rotated environment may not be helpful for improving task performance, especially in undamaged motor systems, since the rotated environment leads to modification of the environment adaptation module but not the task generation module. This is directly seen in the human data for experiment 3, in which the motor task remained the same under all conditions. Performance transiently worsened when the rotation returned to baseline, even though no change in hand movement was required to achieve correct performance. In the simulation model, this occurs because there are two different types of error that are used to train the two learning modules. When the baseline rotation is restored in experiment 3, the performance error e _x_, e _y_ is zero, since the initial hand movement is correct. However, the plant inverse error, e _p_, is nonzero and thus the plant inverse learns (and motor performance changes) even though there was no performance error. This is an important distinction between the two systems. Task learning is driven by performance error, while environment adaptation is driven by predictions of the environment response, independent of the desired task. Therefore this model suggests that virtual reality adaptation may be insufficient to train task performance.

Our results are consistent with the common observation that a skill can often be performed in a different environment with substantially less retraining than originally required to learn the skill, described by Krakauer and colleagues as task-specific savings ( Krakauer *et al.*, 2005). Our results are also consistent with the observation of transient aftereffects after changing the mechanics of the environment. Our results are also consistent with the ability to learn multiple new skills without forgetting previously-learned skills. Although previous models have addressed skill learning or environment adaptation separately, our results and model simulations represent one of the first quantitative studies to examine their interaction. A recent study ( Mazzoni & Krakauer, 2006) on rotational field experiments concluded that implicit adaptation to a visuomotor rotation overrides the explicit strategies given by the experimenters. Despite the use of explicit cognitive strategies that opposes the visual rotation, experiment participants unconsciously adapted to the rotational field, making increasing errors to the target. The rate of adaptation was the similar with and without the explicit cognitive strategies, showing that implicit adaptation occur independent of the use of explicit strategies. The “implicit adaptation” is equivalent to the environment learning network in our model, and “explicit strategies” are equivalent to task learning in our model. Their results are consistent with our model, showing that two types of learning interact for visuo-motor adaptations. Our model provides a good framework to capture these two types of sensory-motor learning.

Our two-systems model was also consistent with the fast and slow adapting systems as Shadmehr and colleagues proposed ( Smith *et al.*, 2006). The two-rate learning model has been used to explain task interference ( Sing & Smith, 2010), generalization ( Tanaka *et al.*, 2012), savings ( Zarahn *et al.*, 2008) and retention ( Joiner & Smith, 2008). We believe that our two system model will offer another perspective in differentiating the two learning systems. Our environment adaptor would behave similarly to the fast adapting system, and the trajectory generator would behave like the slow adapting system.

There are several weaknesses of the current model that need to be addressed in future experiments. The model does not explain the observation that learning two very similar skills can generate interference. Such an observation could be incorporated in a model in which different tasks are represented not by a set of discrete motor programs but by a parameterized or “fuzzy” mixture of motor programs perhaps using a local basis function network ( Poggio & Girosi, 1990). The model also does not explain the observation that after extensive practice it is possible to switch between two environments (e.g. prism glasses) almost instantly ( Martin *et al.*, 1996; Shadmehr & Wise, 2005). This observation could be incorporated using an environment learning model that can learn to respond to cues indicating a change in the environment. Our model does not yet explain differences in performance following blocked or interleaved practice ( Simon & Bjork, 2001). This would depend upon the details of the task learning and environment adaptation algorithms. For instance in certain neural network algorithms, blocked practice (as in our experiment) might be more likely to retrain existing weights to fit the most recent condition, while interleaved practice might be more likely to fit the network output so that it performs correctly in multiple different conditions. Furthermore, our models were limited to using errors in the visual domain and did not capture performance measures and errors in other domains important to motor performance such as proprioception. Incorporation of multi-dimensional sensory information is needed to fully capture human motor learning and to explain behaviors in proprioception-driven tasks.

The two learning systems proposed in the model are analogous to two types of control systems. The task learning system can be compared to an optimal controller that learns a desired trajectory that will achieve the task goal. The environment learning system can be compared to an adaptive controller that learns the motor commands required to achieve the desired trajectory in the current environment. This is similar to the differing neuro-anatomical modules in Doya’s proposed framework ( Doya *et al.*, 2001). In this context, it is interesting to speculate whether errors in the adaptive controller could facilitate or interfere with learning in the optimal controller. If so, then it might be possible to use our model to redesign current virtual-reality training programs so that a change in the environment that leads to a change in the adaptive controller might also facilitate task learning. In future studies we plan to study this potential interaction, and we plan to investigate the interaction of the two learning systems for more complex tasks in which the dynamics of movement must be learned.

### Informed consent

Participants signed written consent for the experiment and HIPAA authorization for the use of personal data.

### Data availability

The data referenced by this article are under copyright with the following copyright statement: Copyright: © 2014 Chu VWT and Sanger TD

Data associated with the article are available under the terms of the Creative Commons Zero "No rights reserved" data waiver (CC0 1.0 Public domain dedication). 

*figshare*: Data from motor learning experiments, doi: 10.6084/m9.figshare.957526 ( Chu & Sanger, 2014).
