# Supplementary material for: Two different motor learning mechanisms contribute to learning reaching movements in a rotated visual environment
Source: F1000Res. 2014 Dec 10;3:72. Originally published 2014 Mar 17. [Version 2] doi: 10.12688/f1000research.3676.2 (PMC4670007; doi:10.12688/f1000research.3676.2)
Supplement: Peer review history

# Peer review: Referee response for version 2

**DOI:** 10.5256/f1000research.6149.r11102

**Published:** 2015-11-23

**Authors:** Eileen Lew

**Affiliations:** 1 Faculty of Engineering, Computing and Science, Swinburne University of Technology-Sarawak, Kuching, Malaysia

The paper has been revised based on the concerns addressed by previous reviewers where clarity has improved greatly. A few minor points as follows:

1. The title could be improved to highlight differences in transfer of learning from artificial to natural environment instead of focusing only about rotated visual environment (artificial environment), as the title appears misleading. The use of the keywords, such as environment adaptation and skill learning could better highlight the content and outcome of the paper as author has done extensive analysis on the interactions of the two environments.
2. There is no mention about the follow-up study (8 participants) in the abstract, which is could be interesting to put forward the intention to study generalization and the outcome.
3. In terms of future work, as this study focused on simple task, the next question will be on complex task. Authors could elaborate further on the hypothesis of the learning systems for more complex tasks and proposition on how the dynamics of the movements can be learned. 

I have read this submission. I believe that I have an appropriate level of expertise to confirm that it is of an acceptable scientific standard.

# Peer review: Referee response for version 2

**DOI:** 10.5256/f1000research.6149.r7050

**Published:** 2015-02-24

**Authors:** Rajiv Ranganathan

**Affiliations:** 1 Department of Kinesiology, Michigan State University, East Lansing, MI, USA

The authors have satisfactorily addressed my concerns from the previous review. I only have a couple of minor points:

1. It seems that the same 10 participants were used in all 4 experiments (and a further 8 were used for bigger angles). Was the order of the experiments randomized or did participants do the experiments in sequence? Also, what was the time gap between experiments? This detail could be included under the "Participants" section.
2. There seems to be a problem in Figure 3D - On the monitor, the movements in A and C are not aimed toward the target

I have read this submission. I believe that I have an appropriate level of expertise to confirm that it is of an acceptable scientific standard.

# Peer review: Referee response for version 1

**DOI:** 10.5256/f1000research.3940.r5806

**Published:** 2014-09-09

**Authors:** Rajiv Ranganathan

**Affiliations:** 1 Department of Kinesiology, Michigan State University, East Lansing, MI, USA

**Summary:**

The goal of this paper by Chu and Sanger is to examine the hypothesis that learning mechanisms that adapt to altered environments (like visuomotor rotation) is different from learning mechanisms that are required for improvement of motor skills.  Using both experiments and model simulations, the authors make the argument that there are indeed two learning mechanisms --  a learning mechanism for each task (which accounts for the specificity of learning of different skills) but a single learning mechanism for learning the environment (which accounts for generalization within a single environment).

The topic addressed in the paper is of great interest to readers in the motor control community. Overall the paper is well-written and the experiments/modeling are well done.

**Major concerns:**

1. The paper does not seem to make reference to a very similar idea of “model-based” and “model-free” learning ( Huang *et al., *2011, Haith & Krakauer, 2013). Those papers make almost the exact same distinction where model-based learning accounts for tasks like adaptation, whereas model-free mechanisms account for “skill learning” tasks.  It would be useful for the authors to look at this distinction and discuss these ideas in relation to the current paper.
2. Huang *et al.* (2011) also used a similar paradigm to Experiment 3 to show that practicing in an “opposite” visuomotor rotation could lead to savings (faster re-learning) if the hand-direction was the same (due to model-free learning). Therefore, the authors may want to use caution before making statements such as “adaptation to an altered environment is not useful for training task performance”.
3. While the statistics are used in a “within-subject” kind of way with the z-statistic, I have a couple of concerns:

- The sample standard deviation is estimated from only 10 trials  - therefore the use of a t-distribution may be more appropriate than a z-distribution.
- The sign of the after-effect is masked by this analysis – i.e. both undershoots and overshoots would count as being significant since only the absolute value is being taken into account.
- It would therefore be useful to do a typical "between-subjects" analysis (like a paired t-test) which would also help the reader know the actual magnitudes of the after-effects across the 4 experiments (and not merely whether they were significant or  not). 

**Minor:**

1. I do not understand how the two stripes are representing “West”. Does one stripe represent West in visual space, and the other in hand space? If so, how did the participants distinguish the two stripes? Also it seems that in Figure 3B, the middle panel “Interference:B1” has the directions marked incorrectly.
2. Figure 2 is a little hard to digest and has repetition of the same blocks across multiple panels – it would help the readers if the authors could omit the equations (or use them just once if they are repeated) so that readers could clearly grasp the critical differences between the 3 models.
3. The rationale behind Experiment 4 is a little unclear to me. What do the authors mean when they say “we had to change the visual display”(do they mean target position?). There also seems to some discrepancy between Figure 3D and the table in Figure 3 (e.g. figure 3D should show Baseline "A2" and Readaptation A2, not A1). Also the titles for each experiment condition in Figures 3A-D are all labeled the same "Baselne A1 Interference B1 and Re-Adaptation A1".
4. The lesser strength of effects in the 90° experiments could be explained by different strategies used in small versus large visuomotor rotations ( Abeele & Bock, 2001).
5. The ideas in the paper would also be helped by reference to older ideas of “task and practice specificity” of motor skills (Henry, 1959) – which suggest poor generalization of motor skills.
6. It would help to line up the experimental data in Figure 5 alongside  the model predictions in Figure 4 so that it is easier to identify the best predicting model.
7. Why is there a difference in the learning rate between the 3 models during adaptation to the first task (Figure 4, Experiment 1 trials 1-20)? Is it a fair comparison among the 3 models if the final performance of all models is not equivalent after this first block?
8. Since Experiment 3 is the real important finding that confirms the author's model of motor learning, it would help to show in a Figure the fact that participants actually increase the error in the first few trials (currently it is not possible to see this clearly because all 60 trials are all compressed into the figure).

I have read this submission. I believe that I have an appropriate level of expertise to confirm that it is of an acceptable scientific standard, however I have significant reservations, as outlined above.

# Response

**Published:** 2014-12-01

**Authors:** Virginia Chu

**Affiliations:** University of Illinois at Chicago, USA

Thank you for your suggestion on additional discussion areas and statistical analysis. We have addressed your comments in our second version with the details highlighted below.

1. Thank you for your suggestion with the discussion on model-based and model free learning models, we have added a discussion on this topic in the manuscript.
2. Regarding the use of z-statistics as a measure in our study, we agree that this is not a conventional way of using this measure. What we call “z” is using a measure similar to the z-statistics, since our sample size was 1 (aftereffect trial) to be compared with a population size of 10 (baseline). In using a z-statistics-like measure, this allows us to use the baseline mean and standard deviation of the baseline trials as the population mean and standard deviation. However, if we use the t-statistics, it will require that we have the sample’s standard deviation (which is 0 for a sample size of 1). Therefore, we chose to use the z-statistics as the basis of for our statistical measure.
3. In order to show the sign of the after effect for readers to examine, we have changed the reported z value to be without the absolute value, and use |z| for statistical comparison. As shown in the new Table 1 and 2, only 3 values were affected, all insignificant z values for Experiment 2.
4. As requested by both reviewers, regarding information for typical “between-subject” analysis, we performed additional statistical analysis and they are reported in the text and in the new figures 5 and 7.
5. Thank you for pointing out the error in the notation in figure 3B. We have corrected that. The 2 stripes were used as 1 symbol. They are together used as an indicator for changes in the rotation environment. They were deliberately vague so that the participants would notice a change, but cannot clearly determine the amount of visual rotation.
6. We made Figure 2 simpler and changed the shading of the border markings to make the models easier to read.
7. Figure 3 has been corrected. We apologize for the mislabeling. We have also clarified the rationale for experiment 4 to make things clearer.
8. Thank you for pointing out two relevant references (Abeele & Bock, 2001) and (Henry, 1959). Unfortunately, we were not able to locate (Henry, 1959). Were you by chance referring to (Henry, 1958/1968)? We have added a discussion on the topics in the introduction and discussion.
9. We agree that comparison between Figure 4 and 5 is important in the identification of the best predicting model. Unfortunately, we feel that putting the subject data from Figure 5 within Figure 4 would make the figures too small to give. Instead, we re-arranged the order of the graphs in Figure 5, to facilitate comparison between the two figures.
10. The model simulations are meant for qualitative comparisons. Thank you for pointing out that the learning rate and final performance in the end of the first block was not the same. Our primary focus was to keep the model components to be as consistent as possible across the 3 models, so that the only difference between the models is the model structure. As an example, each learning network in the controller and the time update in the online correction (Figure 2) for all 3 models used the same model parameters. The differences observed in the simulation results, simply result from the difference in model structure. We felt that this was a more important comparison to examine from the simulation results, rather than tuning the model parameters so that the learning rates and final performance to be equal. It is important to note that the difference in simulation results stemmed simply from a difference in model structure using the exact same model components and parameters. Model C appear to have higher learning rate because it has an additional learning component (environment adaptation controller) compared to model A and B. Model A and B could be made to have the same learning rate and final performance in the baseline block by tuning λ, the learning rate of the system (using a faster rate). But this would not change the pattern of results we see in the simulation, and it is more important to us that the model parameters were kept the same.

In order to demonstrate the errors in Experiment 3 more clearly, the trajectory errors were shown in Figure 6, where the last trial of baseline and the first trial of the remaining 2 blocks were shown. Further, the errors were also show numerically in Table 1.  With the new Figure 5E and Figure 7E, the group means of the errors in the first trial in state C are now shown clearly in a bar chart form.

# Peer review: Referee response for version 1

**DOI:** 10.5256/f1000research.3940.r5345

**Published:** 2014-07-04

**Authors:** Gelsy Torres-Oviedo

**Affiliations:** 1 Departments of Bioengineering, University of Pittsburgh, Pittsburgh, PA, USA

The title is appropriate for the work. The abstract needs to be revised to improve its clarity. Specifically, the authors need 1) to include a brief definition for skill learning and 2) to clarify that after-effects equate to poor skill performance in their study since literature presents some other cases of after-effects leading to improved movements.Overall the article content is clearly explained and very well written. There are only a few general conceptual points that should be addressed. Authors should consider revising their conclusions based on these points.

1. It is unclear how different the reaching directions need to be, for them to be considered the same tasks or skills?
2. A common patients' deficit is their perseverant behavior from one situation to another (even when this is inappropriate). Literature shows this motor perseverance appears beneficial when experiencing environmental adaptations. Given that patients continue doing the adapted motion when the external perturbation is removed, it ultimately leads to long-term changes in their movements with repeated exposure. The authors are very adamant in their conclusions that environmental adaptation cannot be used as a rehabilitation intervention, yet their two-mechanism model does not seem to include this perseverance feature of patients' behavior. Kindly elaborate on this.
3. In their theoretical framework, authors present a single performance error and they do not seem to differentiate between self-generated and externally generated performance errors. Possibly this simplification limits their model to predicting behaviors during visual tasks but it would fail at explaining behaviors in proprioception-driven tasks, like reaching in a force field or walking on a split-belt treadmill.

Other comments in order of appearance:

- Page 4: Link description of model predictions to their visual representation on Fig 4.
- Conceptual question in Figure 2: It is not clear why the desired trajectory generator is not part of the controller block in which all the trajectories are generated.  What are the learning network blocks computing? 
- Illustration points in Figure 2:
   
  - The "environment adaptation module" in Model C could be better indicated, since it is not very clear that it is missing in Model A and B.
  - The equations for the online correction on Fig. 2 do not match the ones on the text (page 7). Kindly add V and V' on the text or change equations on Fig. 2.
- Page 6: x _c_' and y _c_' are missing the c.
- Page 6, under Trajectory generator section: It is not well explained what j and i are on the equations.
- Page 6 under environment adapter section: Authors should consider adding the equation in which x _c_' and y _c_' = Phat ^-1^ [x _c_ y _c_] and the learning rule for P, as they do for indicating the update of W.
- Page 7: Authors randomize the order of the 4 experiments to account for the order of the task. However, it is not very clear if repeated exposures of the adaptation task changes subjects behaviour.
- Page 7, under procedure: In the explanation of experiment 4 it is unclear if subjects were able to see the cursor or not.
- Page 7: Authors equate the intended movement to the target position. However, the intended movement could be different from the target position when subjects are told to aim at a different direction (as done by Mazzoni and Krakauer, 2006). Please clarify.
- Page 9, under data analysis section: Authors perform z-score to determine if after-effects are statistically different from baseline. It is unclear whether similar results are obtained if means (baseline vs after-effect) are compared.  

I have read this submission. I believe that I have an appropriate level of expertise to confirm that it is of an acceptable scientific standard, however I have significant reservations, as outlined above.

# Response

**Published:** 2014-12-01

**Authors:** Virginia Chu

**Affiliations:** University of Illinois at Chicago, USA

Thank you for your valuable comments and we have addressed them in our new revision in detail, listed below.

1. Thank you for your suggestion on the abstract. It has been edited accordingly.
2. You have raised a very interesting topic on the delineation between same and different tasks/skills. As there is no consensus in the scientific community regarding the criteria for defining tasks that are the same or different, this provides for an interested discussion. We have added a discussion on this topic
3. We agree with your assessment of patient deficits. Our simulation focused on understanding typical adult motor performance, and thus did not address motor performance in motor disorders. We believe that the type of perseverant behavior in patients can be also understood in our model frameworks. We have added a discussion on this topic.
4. Thank you for pointing out a limitation of our simulation. Although we did not clearly delineate the two errors as you have categorized them, we believe that we were able to capture some of that information. In models A and B, there was only a single performance error (difference between performance and desired trajectory). However, in model C, we had two different performance errors that drive the two networks: difference between performance and desired trajectory to drive the trajectory controller, and the difference between performance and the expected output to drive the plant estimator. Using your terminology, we believe that the trajectory error can provide information about self-generated errors, and the plant estimation error can give us information about externally generated performance errors. But since our models were not meant to simulate proprioceptive tasks, we do believe that further adjustments to the models will be needed to properly simulate proprioceptive tasks. A discussion has been added to the limitations.
5. Instead of adding the link between model predictions on page 4 to Figure 4, we have added a description in the results section. Since the experiments were not yet described in page 4, it may be premature to link to Figure 4 at that part of the manuscript.
6. In regards to Figure 2, whether the desired trajectory generator was included in the controller block was only a matter of notation. We chose not to include that in the controller block because no learning occurred in the desired trajectory generation as the desired trajectory was programmed to be a straight line with bell shape velocity from start to end point. The learning network blocks performed the computation for the neural networks described in paragraph 2 and 3 of “Trajectory generator” section of the Simulations.
7. We have updated Figure 2 to better indicate the environment adaptation module. In the text, we have combined the equations for online correction into one equation causing the confusion of the equation mismatch. We have changed the text to reflect the same notations used in the figure.
8. Page 6: Thank you for pointing out the missing c, which has been corrected. The i and j are dummy variables for the summations, we have added clarification for this in the text. The learning rule for P was shown as the equation with delta P. The equation indicated the output has been added for clarity.
9. Regarding the random order of experiments, we did not believe that the repeated exposure of the adaptation task changed subject behavior as the experiment was relatively short compared to most adaptation studies (20 trials per block). The randomization was used to safe guard against any potential adaptation towards the end of the experiment. After the experiment, we did not see any overall patterns of behavior change according to experimental order.
10. The cursor was visible to participants under all experimental conditions during the reach.
11. For the purpose of our study, the intended movement and the target position is essentially the same as long as the subjects were following our instructions. As you pointed out, there are circumstances where there may be disagreements between the two terms such as in Mazzoni and Krakauer paper where they examined the use of explicit strategies. I would argue that Mazzoni and Krakauer were essentially giving their participants another “invisible” target to aim for as we did for our experiment 4, and in that case, the new invisible target becomes the intended movement. But we agree that the particular sentence in question was poorly structured and had been rewritten for clarity.
12. As requested by both reviewers, we performed additional statistical analysis and they are reported in the text and in the new figures 5 and 7.
